# Supplementary material for: A retrospective study on the evaluation of the appropriateness of oral anticoagulant therapy for patients with atrial fibrillation
Source: PLoS One. 2021 Nov 11;16(11):e0259199. doi: 10.1371/journal.pone.0259199 (PMC8584758; doi:10.1371/journal.pone.0259199)
Supplement: Attachment — Submitted filename: PONE-D-21-18099_reviewer with comments.pdf [file pone.0259199.s002.pdf]

PLOS ONE

REVIEW ORIGINAL RESEARCH CASE REPORT CASE SERIES RAPID A retrospective study on the evaluation of the appropriateness of oral anticoagulant therapy prescription for patients with atrial fibrillation

--Manuscript Draft--

**Manuscript Number:**	PONE-D-21-18099
**Article Type:**	Research Article
**Full Title:**	REVIEW ORIGINAL RESEARCH CASE REPORT CASE SERIES RAPID A retrospective study on the evaluation of the appropriateness of oral anticoagulant therapy prescription for patients with atrial fibrillation
**Short Title:**	evaluation of the appropriateness of oral anticoagulant therapy prescription
**Corresponding Author:**	Jiangya Li<br>Kunming Medical University<br>Kunming, CHINA
**Keywords:**	atrial fibrillation; oral anticoagulants; appropriateness of prescription
**Abstract:**	Purpose<br><br>The purpose of this study was to explore the current differences between clinician prescriptions versus recommended guidelines of oral anticoagulants (OACs) for patients with atrial fibrillation (AF) and to provide directions for the improvement of anticoagulation strategies to treat patients with AF.<br><br>Patients and methods<br><br>Data was collected from the electronic medical records system of Fuwai Yunnan Cardiovascular Hospital between July 2019 and January 2020. Suitability of the the prescribed OACs for patients with AF was evaluated based on «The Rules for Evlauating the Inappropriateness of Prescriptions», the medicine label of the prescription, and any related antithrombotic guidenes for treating patients with AF.<br><br>Results<br><br>A total of 460 patients met the criteria for inclusion. Of those, 56.7% received appropriate prescriptions and 46.3% received inappropriate prescriptions. Of the patients that received inappropriate descriptions, 17.4% were prescribed medication without the presentation of appropriate indicators, 11.1% were prescribed the inappropriate selection of medication, and 29.6% were prescribed the inappropriate dosage of medication. For patients that were prescribed medication without the presentation of appropriate indicators, 12.5% presented no indication for medication and 87.5% presented with indication for medication but not specifically OAC. For patients prescribed with an inappropriate selection of medication, there were 1, 5 and 45 patients taking rivaroxaban, dabigatran and warfarin respectively. The distribution of NOAC dose was as follows: dabigatran standard-dose (43.2%), low-dose (52.3%). rivaroxaban standard-dose (57.5%), low-dose (35.9%), high-dose (4.2%). A total of 44 patients (9.6%) had bleeding events after medication, 12 patients (2.6%) experienced embolic events, and 7 patients had other adverse events.<br><br>Conclusion<br><br>In clinical practice, it is common for patients with AF to receive inappropriate prescriptions of OACs. Therefore, it is necessary to strengthen the anticoagulation management of patients with AF to improve the rational use of OACs.
**Order of Authors:**	Yiyi Qian
	Zhiying Weng
	Jiajun Zhang

Powered by Editorial Manager® and ProduXion Manager® from Aries Systems Corporation

	Jiangya Li
	Shanshan Li
	Liju Liang
**Additional Information:**	
**Question**	**Response**
**Financial Disclosure**<br><br>Enter a financial disclosure statement that describes the sources of funding for the work included in this submission. Review the submission guidelines for detailed requirements. View published research articles from *PLOS ONE* for specific examples.<br><br>This statement is required for submission and **will appear in the published article** if the submission is accepted. Please make sure it is accurate.<br><br>**Unfunded studies**<br>Enter: *The author(s) received no specific funding for this work.*<br><br>**Funded studies**<br>Enter a statement with the following details:<br>• Initials of the authors who received each award<br>• Grant numbers awarded to each author<br>• The full name of each funder<br>• URL of each funder website<br>• Did the sponsors or funders play any role in the study design, data collection and analysis, decision to publish, or preparation of the manuscript?<br>• **NO** - Include this sentence at the end of your statement: *The funders had no role in study design, data collection and analysis, decision to publish, or preparation of the manuscript.*<br>• **YES** - Specify the role(s) played.<br><br>* typeset	YES-Correspondence
**Competing Interests**<br><br>Use the instructions below to enter a competing interest statement for this submission. On behalf of all authors, disclose any competing interests that could be perceived to bias this	The authors have declared that no competing interests exist.

work—acknowledging all financial support and any other relevant financial or non-financial competing interests.<br><br>This statement is required for submission and **will appear in the published article** if the submission is accepted. Please make sure it is accurate and that any funding sources listed in your Funding Information later in the submission form are also declared in your Financial Disclosure statement.<br><br>View published research articles from *PLOS ONE* for specific examples.<br><br>**NO authors have competing interests**<br><br>Enter: *The authors have declared that no competing interests exist.*<br><br>**Authors with competing interests**<br><br>Enter competing interest details beginning with this statement:<br><br>*I have read the journal's policy and the authors of this manuscript have the following competing interests: [insert competing interests here]*<br><br>* typeset	
**Ethics Statement**<br><br>Enter an ethics statement for this submission. This statement is required if the study involved:<br><br>• Human participants<br>• Human specimens or tissue<br>• Vertebrate animals or cephalopods<br>• Vertebrate embryos or tissues<br>• Field research<br><br>Write "N/A" if the submission does not require an ethics statement.	This research study was conducted with the approval of the Ethics Committee of Fuwai Hospital Chinese Academy of Medical Sciences and Fuwai Yunnan Cardiovascular Hospital.

General guidance is provided below. Consult the submission guidelines for detailed instructions. **Make sure that all information entered here is included in the Methods section of the manuscript.**<br><br>**Format for specific study types**<br><br>**Human Subject Research (involving human participants and/or tissue)**<br>• Give the name of the institutional review board or ethics committee that approved the study<br>• Include the approval number and/or a statement indicating approval of this research<br>• Indicate the form of consent obtained (written/oral) or the reason that consent was not obtained (e.g. the data were analyzed anonymously)<br><br>**Animal Research (involving vertebrate animals, embryos or tissues)**<br>• Provide the name of the Institutional Animal Care and Use Committee (IACUC) or other relevant ethics board that reviewed the study protocol, and indicate whether they approved this research or granted a formal waiver of ethical approval<br>• Include an approval number if one was obtained<br>• If the study involved *non-human primates*, add *additional details* about animal welfare and steps taken to ameliorate suffering<br>• If anesthesia, euthanasia, or any kind of animal sacrifice is part of the study, include briefly which substances and/or methods were applied<br><br>**Field Research**<br><br>Include the following details if this study involves the collection of plant, animal, or other materials from a natural setting:<br>• Field permit number<br>• Name of the institution or relevant body that granted permission	
**Data Availability**<br><br>Authors are required to make all data underlying the findings described fully	Yes - all data are fully available without restriction

Powered by Editorial Manager® and ProduXion Manager® from Aries Systems Corporation

available, without restriction, and from the time of publication. PLOS allows rare exceptions to address legal and ethical concerns. See the PLOS Data Policy and FAQ for detailed information.<br><br>A Data Availability Statement describing where the data can be found is required at submission. Your answers to this question constitute the Data Availability Statement and **will be published in the article**, if accepted.<br><br>**Important:** Stating 'data available on request from the author' is not sufficient. If your data are only available upon request, select 'No' for the first question and explain your exceptional situation in the text box.<br><br>Do the authors confirm that all data underlying the findings described in their manuscript are fully available without restriction?	
**Describe where the data may be found in full sentences. If you are copying our sample text, replace any instances of XXX with the appropriate details.**<br><br>• If the data are **held or will be held in a public repository**, include URLs, accession numbers or DOIs. If this information will only be available after acceptance, indicate this by ticking the box below. For example: *All XXX files are available from the XXX database (accession number(s) XXX, XXX.*).<br>• If the data are all contained **within the manuscript and/or Supporting Information files**, enter the following: *All relevant data are within the manuscript and its Supporting Information files.*<br>• If neither of these applies but you are able to provide **details of access elsewhere**, with or without limitations, please do so. For example:<br><br>*Data cannot be shared publicly because of [XXX]. Data are available from the XXX Institutional Data Access / Ethics*	All relevant data are within the manuscript and its Supporting Information files.

Committee (contact via XXX) for researchers who meet the criteria for access to confidential data.<br><br>*The data underlying the results presented in the study are available from (include the name of the third party and contact information or URL).*<br>• This text is appropriate if the data are owned by a third party and authors do not have permission to share the data.<br><br>* typeset	
Additional data availability information:	

Powered by Editorial Manager® and ProduXion Manager® from Aries Systems Corporation

Click here to access/download;Manuscript;A retrospective study.doc

REVIEW   ORIGINAL RESEARCH   CASE REPORT   CASE SERIES   RAPID

A retrospective study on the evaluation of the appropriateness of oral anticoagulant therapy prescription for patients with atrial fibrillation

Yiyi Qian[1¶], , Jiajun Zhang[2¶], Jiangya Li[2¶],Zhiying Weng[2*],Shanshan Li[2&], Liju Liang[2&]

[1]Dept.of Pharmacy, Fuwai Yunnan Cardiovascular Hospital, Kunming, Yunnan, China ;

[2]School of Pharmaceutical Science & Yunnan Key Laboratory of Pharmacology for Natural Products, Kunming Medical University, Kunming, Yunnan, China

Correspondence: Zhiying Weng

School of Pharmaceutical Science & Yunnan Key Laboratory of Pharmacology for Natural Products, Kunming Medical University, Kunming, Yunnan, China

Tel 13888320577

Email weng_zy@sina.com

Abstract:

Purpose: The purpose of this study was to explore the current differences between clinician prescriptions versus recommended guidelines of oral anticoagulants (OACs) for patients with atrial fibrillation (AF) and to provide directions for the improvement of anticoagulation strategies to treat patients with AF.

Patients and methods: Data was collected from the electronic medical records system of Fuwai Yunnan Cardiovascular Hospital between July 2019 and January 2020. Suitability of the the prescribed OACs for patients with AF was evaluated based on «The Rules for Evlauating the

Inappropriateness of Prescriptions», the medicine label of the prescription, and any related antithrombotic guidenes for treating patients with AF.

Results: A total of 460 patients met the criteria for inclusion. Of those, 56.7% received appropriate prescriptions and 46.3% received inappropriate prescriptions. Of the patients that received inappropriate descriptions, 17.4% were prescribed medication without the presentation of appropriate indicators, 11.1% were prescribed the inappropriate selection of medication, and 29.6% were prescribed the inappropriate dosage of medication. For patients that were prescribed medication without the presentation of appropriate indicators , 12.5% presented no indication for medication and 87.5% presented with indication for medication but not specifically OAC. For patients prescribed with an inappropriate selection of medication, there were 1, 5 and 45 patients taking rivaroxaban, dabigatran and warfarin respectively. The distribution of NOAC dose was as follows: dabigatran standard-dose (43.2%), low-dose (52.3%). rivaroxaban standard-dose (57.5%), low-dose (35.9%), high-dose (4.2%). A total of 44 patients (9.6%) had bleeding events after medication, 12 patients (2.6%) experienced embolic events, and 7 patients had other adverse events.

Conclusion: In clinical practice, it is common for patients with AF to receive inappropriate prescriptions of OACs. Therefore, it is necessary to strengthen the anticoagulation management of patients with AF to improve the rational use of OACs.

Keywords: atrial fibrillation, oral anticoagulants, appropriateness of prescription

Introduction

[1] Atrial fibrillation (AF) is the most common arrhythmia encountered in clinical practice and can result in strokes, one of the most common and severe complications of AF. [2] AF increases the risk of stroke five-fold. [3] Stroke has the characteristics of high disability, high mortality and high incidence. [2] Furthermore, it imposes a significant burden on healthcare systems, patients and their families.

[4] Anticoagulation therapy is the cornerstone for the prevention of stroke in patients with AF. [5] With the widespread use of new oral anticoagulants (NOACs) in clinical practice in recent years,

there are many choices of anticoagulation drugs for patients with AF, and the quality of anticoagulation is variable. Most patients with AF have not received standardized anticoagulation therapy.[6,7] In practice, the prescription rate of oral anticoagulants (OACs) is low and many patients with AF or a previous history of stroke have not received appropriate anticoagulation therapy. Furthermore, the choice of anticoagulation drugs in many prescriptions is irrational with poor anticoagulation control and inappropriate usage and dosage of OACs. This increases the occurrence of adverse events such as thromboembolism and bleeding. [8] A subgroup analysis of Chinese patients in the GARFIELD study showed that only 28.7% of the patients receive anticoagulation treatment, of which 22.2% received warfarin therapy, and 6.5% received NOACs. 19.8% of patients had not received any anticoagulation therapy, and 51.6% of patients received antiplatelet drugs alone.[9] Globally, half of all newly diagnosed AF patients at low risk are receiving OACs unnecessarily, which increases their risk of bleeding. [10] Franchi C et.al. assessment of the appropriateness of OACs prescriptions and its associated factors in hospitalized patients aged 65 years or older showed that nearly 44% of patients were inappropriately prescribed OACs. Most patients were underprescribed or prescribed an inappropriate antithrombotic drug.

[7] With the increasing prevalence of AF, prescription of the appropriate OACs according to each patients' characteristics becomes a challenge. Improving clinicians' prescriptions of OACs for stroke prevention in patients with AF will become a potential area of focus in future interventions.[11,12] This study evaluates the prescription suitability of OAC prescriptions [Warfarin and NOACs (dabigatran and rivaroxaban)] for patients with AF in Fuwai Yunnan Cardiovascular Hospital using the guidelines for AF and the relevant information on prescribed drug labels. By exploring the differences between current prescriptions by clinicians and the guideline recommendations for OACs for patients with AF, this study aims to provide insights for the improvement of anticoagulation strategies for patients with AF.

Material and methods

study design

This research is a single-center, retrospective, cross-sectional real-world study. All patients diagnosed with AF admitted to the Fuwai Yunnan Cardiovascular Hospital between July 2019, and January 2020 were included. The inclusion criteria of the present study were as follows: ①All patients were diagnosed with AF by clinical manifestations, physical examination and electrocardiogram (including patients who are currently receiving or had received anticoagulation therapy). ②Age > 18 years. Exclusion criteria are as follows: transient factors include patients with AF caused by hyperthyroidism, acute pulmonary embolism, recent major surgery or acute myocardial infarction.

Data collection

Data was derived from the electronic medical records system of Fuwai Yunnan Cardiovascular Hospital. The following basic data were recorded for each patient from the medical records: demographic characteristics (sex, age, weight, smoking history, bleeding history, etc.), comorbidity conditions (congestive heart failure, hypertension, diabetes, vascular diseases, etc.), laboratory examinations [serum creatinine, Hemoglobin, alanine aminotransferase (ALT), aspartate aminotransferase (AST), total bilirubin (TBIL), left ventricular ejection fraction (LVEF)], combined medication (aspirin, clopidogrel, amiodarone, verapamil, digoxin, proton pump inhibitors, etc.). The CHA2DS2-VASc score, HAS-BLED score and SAMe-TT2R2 score were calculated for all patients (Table 1-3 for details).

Table 1 CHA2DS2-VASc score[2]

Risk factors	Points awarded
Congestive heart failure	1
Hypertension	1

Age ≥ 75	2
Diabetes mellitus	1
Stroke (Previous stroke, TIA or Thromboembolism)	2
Vascular disease	1
Age 65-74 years	1
Sex category (female)	1

TIA- : transient ischaemic attack.

Table 2 HAS-BLED score[2]

Risk factors	Points awarded
Uncontrolled hypertension	1
Abnormal renal and/or hepatic function	1 point for each
Stroke	1
Bleeding history or predisposition	1
Labile INR	1
Aged >65 years or extreme frailty	1
Drugs or excessive alcohol drinking	1 point for each

INR-: international normalized ratio.

Table 3 SAMe-TT2R2 score[2]

Risk factors	Points awarded
Sex (female)	1
Age <60	1
Medical history of ≥ 2 comorbidities [hypertension, diabetes mellitus, CAD/myocardial infarction, PAD, Chronic heart failure, previous stroke, pulmonary disease, and hepatic or renal disease]	1
Treatment (interacting drugs, e.g. amiodarone)	1
Tobacco use	2

Race [non-Caucasian]	2

CAD-: coronary artery disease; PAD-: peripheral artery disease;

Evaluation criteria for prescription suitability

[11,12] The suitability of OACs for patients with AF were assessed based on the rules for evaluating the inappropriateness of prescriptions in “Guidelines for the Management of Hospital Prescription Reviews (Trial)” (Weiyiguanfa [2010] No. 28) issued by the National Health Commission and the warfarin/rivaroxaban/dabigatran and other related antithrombotic drug labels.

According to the current guidelines, the stroke risk of patients with AF is estimated based on the CHA2DS2-VASc score. OAC is recommended for stroke prevention in AF patients with CHA2DS2-VASc score ⩾ 2 for men or ⩾ 3 for women. OAC should be considered for stroke prevention in AF patients with a CHA2DS2-VASc score of 1 for men or 2 for women. For patients with a single non-sex CHA2DS2-VASc stroke risk factor, we suggest oral anticoagulation over no therapy, aspirin, or combination therapy with aspirin and clopidogrel; antiplatelet therapy should not be used for stroke prevention in AF patients. Also, patients undergoing radiofrequency ablation during the perioperative period (within four weeks after surgery) need to receive anticoagulation therapy. Unless there are anticoagulation contraindications, these patients should not use antithrombotic drugs other than OACs. The combination of OAC and antiplatelet drugs (aspirin or clopidogrel) is considered “appropriate” only within 1-12 months after stent implantation.

The dose of NOACs and the suitability of selected drugs are evaluated based on renal function (creatinine clearance rate, CrCl), age, weight and accompanying interaction drugs. The evaluation criteria are as follows: (1)The conventional dose of dabigatran is 150mg/bid. When the age is ⩾ 80 years or CrCl 30~50ml/min or combined with verapamil, reduce the dosage to 110mg. When between 75~80 years with high bleeding risk (such as HAS-BLED ⩾3 points) or gastritis/esophagitis /Gastroesophageal reflux, dosage can be reduced to 110mg; (2)The conventional dose of rivaroxaban is 20mg QD. When the age is >75 years or CrCl 15-50ml/min,

or the body weight is low, reduce the dosage to 15 mg. The prescribed dose is divided into standard-dose, low-dose and high-dose. Low-dose and high-dose are considered “dose inappropriate”. If patients who do not meet the above criteria for the use of NOACs, then the “selected drugs are not appropriate”. Warfarin is still the only treatment method recognized as safe for patients with valvular AF, and NOACs are prohibited. If patients with valvular AF have prescribed NOACs, they are deemed to be prescribed an “inappropriate selection of drugs”. The guide recommends using the SAMe-TT2R2 score to help identify patients who are unlikely to obtain a good TTR (i.e. SAMe-TT2R2 score >2) and use NOACs with warfarin treatment. If patients with SAMe-TT2R2 score >2 use warfarin but not NOACs, their prescription for “the selection of drugs is inappropriate”.

Also, electronic medical records are retrieved to determine clinical results that may be related to inappropriate medications, such as bleeding events and thromboembolic events, to evaluate the safety of taking OACs in patients with AF.

Ethics Committee

This research study was conducted with the approval of the Ethics Committee of Fuwai Hospital Chinese Academy of Medical Sciences and Fuwai Yunnan Cardiovascular Hospital.

Statistical analysis

Data was collated in Microsoft Excel spreadsheets. The statistical analysis was performed using SPSS17.0, and measurement data were expressed as mean ± standard deviation (SD), Categorical variables were presented as the number of cases (n) and percentages. When investigating the difference between the dose of NOACs and the unreasonable dose, Univariate analyses (t-tests or χ^2 tests) was used to analyze the relevant variables, and significance was judged when $P < 0.05$.

Results

Baseline characteristics

A total of 460 patients were included in this study, and 371 patients were treated by OACs, of which 72 received warfarin, 132 received dabigatran, 167 received rivaroxaban, and a total of 89 patients did not receive anticoagulation therapy. Table 4 summarizes the baseline characteristics of the patients.

Table 4 Baseline characteristics of the study population

	warfarin (n=72)	dabigatran (n=132)	rivaroxaban (n=167)	no OACs (n=89)
age $(\bar{x}\pm s)$, years	65.43±11.862	64.39±12.036	67.5±11.825	64.64±13.179
male	37（51.3%）	79（59.8%）	108（64.6%）	53（59.5%）
atrial fibrillation type				
valvular AF	20（27.8%）	3（2.3%）	1（0.6%）	1（1.1%）
non-valvular AF	52（72.2%）	129（97.7%）	166（99.4%）	88（98.9%）
radiofrequency ablation	10（13.8%）	54（40.9%）	51（30.5%）	2（2.2%）
stent implantation	0	2（1.5%）	9（5.4%）	2（2.2%）
combined disease				
congestive heart failure	16（22.2%）	23（17.4%）	15（8.9%）	10（11.2%）
hypertension	33（45.8%）	76（57.9%）	99（59.2%）	41（46.0%）
diabetes	11（15.2%）	30（22.7%）	27（16.1%）	10（11.2%）
stroke	17（23.6%）	31（23.4%）	55（32.9%）	16（17.9%）
vascular diseases	33（45.8%）	64（48.4%）	107	37（41.5%）

			（64.0%）	
abnormal hepatic function	0	2（1.5%）	1（0.5%）	1（1.1%）
abnormal renal function	1（1.3%）	1（0.7%）	2（1.1%）	1（1.1%）
combined medication				
clopidogrel	3（4.2%）	2（1.5%）	0	13（16.6%）
aspirin	2（2.8%）	0	3（1.8%）	20（22.5%）
aspirin+P2Y12 inhibitor (clopidogrel or ticagrelor）	1（1.4%）	0	4（2.4%）	9（10.1%）

Suitability evaluation of the prescription

From the current analysis, 247 (56.7%) out of 460 patients’ prescriptions were considered appropriate, and 213 (46.3%) were considered inappropriate. Of the patients that received inappropriate descriptions, 80 (17.4%) were prescribed medication without the presentation of appropriate indicators, 51 (11.1%) were prescribed the inappropriate selection of medication, and 136 (29.6%), were prescribed the inappropriate dosage of medication.

For the patients presenting with inappropriate indications, 10 (12.5%) had no indication for medication, and 70 (87.5%) had indication for medication but not OAC. For patients who are indicated for medication but do not use OAC, the most commonly cited reasons by clinicians was worry for a high risk of bleeding. The second most commonly cited reason is that some patients have low compliance and refused anticoagulation medicine.

For the patients with prescriptions of the inappropriate selection of drugs, there were 1, 5 and 45 patients taking rivaroxaban, dabigatran and warfarin respectively. The reasons for these prescriptions were were analysed, as shown in Table 5.

Table 5 Reasons for the inappropriate selection of drugs

reasons for inappropriate	the number of cases

rivaroxaban	rivaroxaban is prohibited for valvular AF	1
dabigatran	dabigatran is prohibited for valvular AF	3
	dabigatran is contraindicated in patients with CrCl<30ml/min	2
warfarin	SAMe-TT2R2 score > 2 points, TTR compliance rate is low	45

Table 6 shows the relevant factors that affect the choice of drug dose. For patients taking dabigatran and rivaroxaban, there were significant differences (P<0.05) in the low-dose group as compared with the standard-dose group in terms of age, stroke risk score, and CrCl. In patients taking rivaroxaban, there was a significant difference in CrCl (P<0.05) when the low dose group was compared with the standard-dose group. No statistical significance in age, stroke and bleeding scores (P>0.05) were found between the low dose and standard-does group in patients taking rvaroxaban.

Table 6 Basic characteristics of NOACs dose

	dabigatran			rivaroxaban			
	standard-dose (n=57,43.2%)	low-dose (n=69,52.3%)	*P*	standard-dose (n=96,57.5%)	low-dose (n=60,35.9%)	high-dose (n=7,4.2%)	*P*
**age**	68.86±12.914	60.61±9.687	0.0001	65.09±12.169	71.52±10.331	70.14±9.19	0.001[a]<br>0.286[b]
<60	16 (28.1%)	34 (49.3%)		34 (35.4%)	7 (11.7%)	1 (14.3%)	
60-69	5 (8.8%)	18 (26.1%)		26 (3.5%)	12 (20.0%)	4 (57.1%)	
70-79	21 (36.8%)	17 (24.6%)		25 (27.1%)	28 (46.7%)	0	
≥80	15 (26.3%)	0		11 (11.5%)	13 (21.7%)	2 (28.6%)	
**CHA2DS2-VASc**	3.65±2.109	2.74±1.578	0.007	3.15±1.886	4.03±1.657	4.43±1.902	0.003[a]

							0.086[b]
0	2 (3.5%)	7 (10.1%)		7 (7.3%)	1 (1.7%)	0	
1	8 (14.0%)	10 (14.4%)		15 (15.6%)	3 (5.0%)	0	
2	11 (19.3%)	9 (13.0%)		17 (17.7%)	5 (8.3%)	2 (28.6%)	
3	7 (12.3%)	22 (31.8%)		16 (16.7%)	13 (21.7%)	0	
≥4	29 (50.9%)	21 (30.4%)		41 (42.7%)	38 (63.3%)	5 (71.4%)	
**HAS-BLED**	2.16±1.279	1.41±0.828	0.0001	1.79±0.962	2.43±0.981	2.43±1.272	0.0001[a]<br>0.101[b]
0	7 (12.2%)	8 (11.6%)		8 (8.3%)	2 (3.3%)	0	
1	12 (21.0%)	32 (46.4%)		30 (31.3%)	6 (10.0%)	2 (28.6%)	
2	12 (21.0%)	22 (31.9%)		34 (35.4%)	24 (40.0%)	2 (28.6%)	
3	17 (29.8%)	7 (10.1%)		22 (22.9%)	21 (35.0%)	1 (14.3%)	
≥4	9 (15.7%)	0		2 (2.1%)	7 (11.7%)	2 (28.6%)	
**CrCl**	63.41±27.66	72.35±19.541	0.036	74.22±25.408	61.73±21.475	41.00±4.203	0.002[a]<br>0.001[b]
>50	33 (57.9%)	64 (92.8%)		81 (84.4%)	43 (71.7%)	0	
30-50	23 (40.4%)	5 (17.2%)		14 (14.6%)	16 (26.7%)	7 (100%)	
15-30	1 (1.8%)	0		1 (1.0%)	1 (1.7%)	0	
**Bleeding event**	7 (12.3%)	4 (5.8%)		5 (5.2%)	14 (23.3%)	1 (14.3%)	
**Thromboembolic events**	2 (3.5%)	4 (5.8%)		2 (2.1%)	3 (5.0%)	0	

Note: a: standard-dose compared with low-dose; b: standard-dose compared with high-dose.

Safety evaluation

Table 7 summarizes the adverse events recorded in the electronic patient case system. A total of 44 patients (9.6%) had bleeding events after medication, including 8, 21, 11 and 4 patients with warfarin, rivaroxaban, dabigatran and non-anticoagulated patients respectively. As shown in Figure 1, rivaroxaban had the highest proportion of bleeding events after medication. A total of 12 patients (2.6%) experienced embolic events, including 5 after taking rivaroxaban, 6 after taking dabigatran, and 1 after taking warfarin. Seven patients had other adverse events with an incidence rate of 1.5%.

Table 7 Adverse events

	adverse events and the number of cases
bleeding events	occult blood (22), subcutaneous hemorrhage (4), gastrointestinal bleeding (3), gingival bleeding (3), fundus bleeding (3), nose bleeding (2), hematuria (2), hemoptysis (1), subdural hematoma (1), urinary tract bleeding (1), ear mucosal bleeding (1), subarachnoid hemorrhage (1)
embolic events	atrial appendage thrombosis (5), recurrent symptoms (4), cerebral infarction (1), coronary embolism (1), ventricular thrombosis (1)
other events	increased digestive tract symptoms, abdominal distension, edema, stomach upset, allergies, dizziness, fatigue and other discomforts

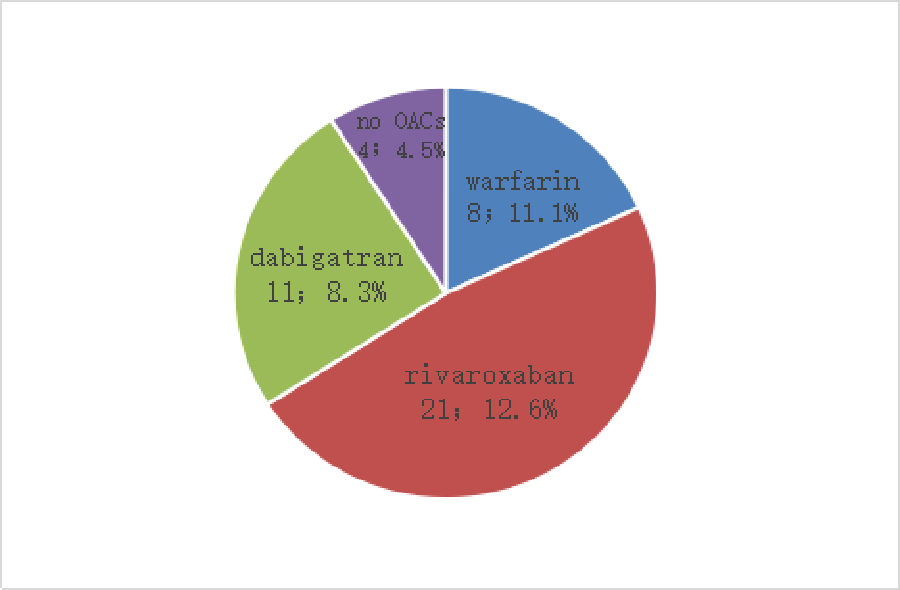

Figure 1 Proportion of bleeding events in patients with AF

Discussion

Dose suitability

In this study, we evaluated the appropriateness and safety of clinicians' presctiptions of OACs for patients with AF. We found that 46.3% of patients were prescribed OACs inappropriately. Of this percentage, the wrong dosage of dabigatran and rivaroxaban was the most common occurrence, accounting for about 29.6%. In particular, an inappropriately low dose was the most common with 52.3 % (69/132) and 35.9% (60/167) of patients being prescribed with low-dose dabigatran and rivaroxaban respectively.

[10,13] This result was consistent with the results reported in previous studies. Their data all show that NOACs have insufficient doses in clinical practice, and most patients did not adjust based on the corresponding reduction indicators but reduced when there was no indication for reduction. This inappropriate reduction could reduce the efficacy of preventing stroke.[14] A retrospective cohort study from Korea showed that when prescribing rivaroxaban for non-moderate renal insufficiency NVAF patients, the combined effect of 20 mg/d is better than 15 mg/d. Similarly,[15] a retrospective cohort study from Taiwan examined the effectiveness and

safety of the recommended dose (15 or 20 mg/d) and low-dose (10 mg/d) prescriptions of rivaroxaban in Asian patients with non-moderate renal insufficiency NVAF ; the results show that compared with the recommended-dose, the low-dose did not significantly reduce the risk of intracranial hemorrhage but increased ischemic stroke. This study does not support the prescription of 10mg/d of rivaroxaban in patients with normal or mild renal insufficiency NVAF.

In our study, the clinicians' specific decision basis for prescribing NOACs was not clear. Analyzing the collected data, we found that the most common reason for patients taking low-dose dabigatran may be due to the dosage packaged by pharmacies. The purchased dose of dabigatran is single with only 110 mg of dabigatran, which limits to some extent the clinician's ability to choose a suitable dose for patients. Secondly, compared to the standard-dose group, we found that the average age, stroke risk, and bleeding risk scores of patients taking the low-dose prescription of dabigatran were lower while their average CrCI was on the high end of the normal range. Also, bleeding events in the low-dose group are relatively low while the incidence of embolism is relatively high. For patients taking rivaroxaban, the average age, stroke risk, and bleeding risk of the low-dose group were higher than that of the standard-dose group while the CrCl was lower than that of the standard-dose group. Both bleeding and embolism events were higher. This suggests the possibility that because anticoagulation is usually a preventive treatment for patients with AF and there are concerns about iatrogenic bleeding events caused by NOACs, clinicians tend to use low-dose NOACs to ensure safety and prevent bleeding. However, the trend of low-dose NOACs may come at the cost of insufficient effectiveness in preventing stroke.

NOACs have predictable pharmacokinetics, fixed-dose regimens. They do not require frequent dose adjustments or routine pharmacodynamic monitoring, but each NOAC has a recommended dose based on clinical characteristics (e.g., age, body weight, renal function, and concomitant medications). To ensure the safety and effectiveness of patients taking NOACs, the prescribing behavior in clinical practice should follow the findings of clinical studies and the instructions on drug labels approved for different NOACs indications in combination with individualised administration to obtain clinical benefit. At the same time, when purchasing drugs,

pharmacies need to fully understand the variation of dosages of dabigatran to meet clinical needs.

Indications suitability

In this study, about 17.4% of patients were prescribed medication without the appropriate indications for medication. The existence of insufficient or excessive anticoagulation was one of the reasons for the prescription. Approximately 12.5% of patients had no indication for medication, and the prescription of medication leads to excessive anticoagulation. The reason for this may be due to the subjective experience of the clinician. About 87.5% of patients presented with indications for medication but did not receive anticoagulant prescriptions, which lead to insufficient anticoagulation. Of these, 52.9% of the patients were at high risk of bleeding. The clinicians caution in preventing the patients' bleeding may have contributed to the lack of anticoagulant prescription. Secondly, 27.1% of patients had poor compliance and refused anticoagulation therapy, which meant these patients used antiplatelet alone to prevent strokes.

Hypertension, age, and stroke history are risk factors for stroke and bleeding in patients with AF that overlap with each other. As the CHA2DS2-VASc score increases, the risk of bleeding increases accordingly. The increased risk of bleeding is often accompanied by an increased risk of embolism. If a patient presents with indications for anticoagulation therapy while their risk of bleeding is also high, it is necessary to conduct a risk and benefit assessment to actively correct the reversible factors contributing to bleeding risk while closely monitoring and formulating an appropriate anticoagulation treatment plan. 16 For patients with a high bleeding risk, the benefits of an appropriate anticoagulation therapy can outweigh the risks, so the risk of bleeding is not necessarily a contraindication to anticoagulation therapy. Furthermore, anticoagulant therapy is the cornerstone of the treatment for patients with AF and antiplatelet therapy alone is not recommended. 17 The BAFTA study showed that compared with warfarin, aspirin has little benefit in terms of anticoagulant therapy in elderly patients with AF. The primary endpoint rate of fatal/disabling stroke (ischemic or hemorrhagic) is 52% higher than that for warfarin while the difference incidence of bleeding events between warfarin and aspirin is not statistically significant.

For patients with AF undergoing radiofrequency ablation, the four weeks following surgery is a high-risk period for the incidence of thrombosis. Thus, regardless of the CHA2DS2-VASc score, anticoagulation must be started and continued for at least four weeks after cardioversion. Whether or not long-term anticoagulation therapy is required after four weeks, should be based on the assessment of the risk for thromboembolism and bleeding. In this study, 2.2% of the patients were in the perioperative period of radiofrequency ablation but were not treated with anticoagulation.

Therefore, to effectively carry out and manage anticoagulation therapy for patients with AF, the proper indications should be correctly grasped in order to determine which patients need anticoagulation therapy. Furthermore, the relevant guidelines and instructions should be strictly followed. Doctors or pharmacists can educate patients about OAC medication to eliminate patients' fears and worries about the bleeding risk and improve patient compliance with medication. Through regular follow-ups, dynamic assessment of embolism and bleeding risk, and close monitoring, clinicians can properly determine when anticoagulation benefits will outweigh the bleeding risk..

Medicine selection

The OACs used in our hospital are mainly warfarin, rivaroxaban and dabigatran. We found that a total of 51 patients were prescribed the inappropriate selection of drugs. Both warfarin and NOACs can effectively prevent strokes in patients with AF. However, compared with warfarin, NOACs can effectively reduce the risk of stroke and systemic embolism while its risk of bleeding is different. At present, 12 the guidelines only recommend NOACs for NVAF. Prescription of NOACs is contraindicated in patients with valvular AF. In this study, four patients with valvular AF were prescribed NOACs, suggesting that the clinicians who implemented the medical order did not grasp which indications were appropriate for the prescription of NOACs. Thus, there may be problems such as inadequate knowledge or clinical evaluation, which potential treatment risks. NOACs are mainly excreted by the kidneys, especially dabigatran. Dabigatran is contraindicated for patients with a CrCl of less than 30ml/min. In this study, two patients with CrCl<30ml/min were

prescribed dabigatran, which was an inappropriate choice. During the use of warfarin, many factors will affect the anticoagulant strength of warfarin. The guide recommends using the SAMe-TT2R2 score to help identify patients who are unlikely to obtain a good TTR through VKA treatment and who will benefit from NOACs more. 18 Studies have shown that the SAMe-TT2R2 score is closely related to TTR in Chinese patients with AF. In this study, 45 patients with SAMe-TT2R2 scores> 2 chose warfarin. These patients need to have increased follow-ups and regular monitoring of coagulation indicators to obtain good TTR during warfarin use. For these patients, NOACs may be a better choice.

Safety

We observed that about 9.6% of patients had bleeding events, and 2.6% had embolism events. In bleeding events, rivaroxaban, dabigatran, warfarin, and non-anticoagulated patients accounted for 12.6%, 8.3%, 11.1% and 4.5% respectively. Among them, occult blood was the most common, followed by digestive tract bleeding. No major bleeding or fatal bleeding occurred. Embolic events were relatively low. We analyzed the dose difference of NOACs through bleeding and embolic events. We found that the bleeding event of the dabigatran standard-dose group was higher than that of the low-dose group, while the embolic event was lower than that of the low-dose group. In contrast, the standard-dose of rivaroxaban had lower bleeding events and embolic events lower than that of the low-dose group. However, since this study did not follow up with patients, it is uncertain whether these safety results are related to inappropriate prescriptions. This should be verified by further research like large-sample prospective studies.

Limitation

This study has certain limitations. Since it was retrospective, not all clinical results are registered and registered results may be biased due to inaccurate or incomplete information. We did not follow up with the patients during the study, and the number of bleeding and thromboembolic events may be underestimated. Also, we did not directly ask the prescribing physicians to determine their method and intention for adjusting the dose, making it difficult to

explain why the inappropriate prescriptions were so. Nevertheless, our data comes from an Asian cardiovascular specialist hospital, and the results are representative enough to illustrate the suitability of OAC prescriptions for patients with AF in this hospital.

Conclusion

Our study found that inadequate anticoagulation and excessive anticoagulation in patients with AF seem to occur frequently and inappropriate dose selection of NOACs is also common. One of the greatest dangers of AF is stroke. To reduce strokes caused by AF, anticoagulation therapy is needed for patients with a high risk of stroke. However, due to insufficient knowledge of the risk assessment of stroke and bleeding in patients with AF, there are still some misunderstandings in clinical anticoagulation therapy for treating AF. To standardize anticoagulation therapy, it is necessary to clarify which patients need anticoagulation therapy as well as the reasonable and standard use of anticoagulant drug dosage. So, while assessing the risk of stroke and bleeding, follow-ups should be increased and changes in clinically relevant risk factors should be monitored during the follow-up period and according to the actual situation of the patient. The complexity of the dose of NOACs depends on related factors such as renal function, age, combination medication. Since NOACs have no corresponding indicators to judge the efficacy, clinicians will feel that using low doses can reduce the bleeding risk, which can result in an insufficient dosage. Therefore, it is necessary to prescribe NOACs in strict accordance with the research findings and medication instructions in clinical practice effectively utilize its anticoagulant effect while avoiding drug-related problems.

Funding

This research was supported by Scientific Research Fund Project of Yunnan Provincial Department of Education (Program No.2018JS245) and Hospital-level scientific research fund project of Fuwai Yunnan Cardiovascular Hospital (Program No.2019YFKT-08).

Disclosure

The author reports no conflicts of interest in this work.

References

1. Rahman F, Kwan GF, Benjamin EJ. Global epidemiology of atrial fibrillation. Nat Rev Cardiol. 2014 Nov;11(11):639-54. doi: 10.1038/nrcardio.2014.118. Epub 2014 Aug 12. Erratum in: Nat Rev Cardiol. 2016 Jul 14;13(8):501.
2. Hindricks G, Potpara T, Dagres N, et al. 2020 ESC Guidelines for the diagnosis and management of atrial fibrillation developed in collaboration with the European Association of Cardio-Thoracic Surgery (EACTS). Eur Heart J. 2020 Aug 29:ehaa612.
3. Chugh SS, Havmoeller R, Narayanan K, et al. Worldwide epidemiology of atrial fibrillation: a Global Burden of Disease 2010 Study. Circulation. 2014 Feb 25;129(8):837-47.
4. Kirchhof P, Benussi S, Kotecha D, et al; ESC Scientific Document Group. 2016 ESC Guidelines for the management of atrial fibrillation developed in collaboration with EACTS. Eur Heart J. 2016 Oct 7;37(38):2893-2962.
5. Lippi G, Mattiuzzi C, Cervellin G, et al. Direct oral anticoagulants: analysis of worldwide use and popularity using Google Trends. Ann Transl Med. 2017 Aug;5(16):322.
6. Pritchett RV, Clarke JL, Jolly K, et al. Clinicians' views and experiences of prescribing oral anticoagulants for stroke prevention in atrial fibrillation: A qualitative meta-synthesis. PLoS One. 2020 May 7;15(5):e0232484.
7. Chang TY, Liao JN, Chao TF, et al. Oral anticoagulant use for stroke prevention in atrial fibrillation patients with difficult scenarios. Int J Cardiol Heart Vasc. 2018 Aug 31;20:56-62.
8. Kakkar AK, Mueller I, Bassand JP, et al. International longitudinal registry of patients with atrial fibrillation at risk of stroke: Global Anticoagulant Registry in the FIELD (GARFIELD).

Am Heart J. 2012 Jan;163(1):13-19.e1.
9. Steinberg BA, Gao H, Shrader P, et al. International trends in clinical characteristics and oral anticoagulation treatment for patients with atrial fibrillation: Results from the GARFIELD-AF, ORBIT-AF I, and ORBIT-AF II registries. Am Heart J. 2017 Dec;194:132-140.
10. Franchi C, Antoniazzi S, Proietti M, et al. Appropriateness of oral anticoagulant therapy prescription and its associated factors in hospitalized older people with atrial fibrillation. Br J Clin Pharmacol. 2018 Sep;84(9):2010-2019.
11. Lip GYH, Banerjee A, Boriani G, et al. Antithrombotic Therapy for Atrial Fibrillation: CHEST Guideline and Expert Panel Report. Chest. 2018 Nov;154(5):1121-1201.
12. January CT, Wann LS, Calkins H, et al. 2019 AHA/ACC/HRS Focused Update of the 2014 AHA/ACC/HRS Guideline for the Management of Patients With Atrial Fibrillation: A Report of the American College of Cardiology/American Heart Association Task Force on Clinical Practice Guidelines and the Heart Rhythm Society. J Am Coll Cardiol. 2019 Jul 9;74(1):104-132.
13. Moudallel S, Steurbaut S, Cornu P, et al. Appropriateness of DOAC Prescribing Before and During Hospital Admission and Analysis of Determinants for Inappropriate Prescribing. Front Pharmacol. 2018 Oct 30;9:1220.
14. Lee SR, Choi EK, Han KD, et al. Optimal Rivaroxaban Dose in Asian Patients With Atrial Fibrillation and Normal or Mildly Impaired Renal Function. Stroke. 2019 May;50(5):1140-1148.
15. Cheng WH, Chao TF, Lin YJ, et al. Low-Dose Rivaroxaban and Risks of Adverse Events in Patients With Atrial Fibrillation. Stroke. 2019 Sep;50(9):2574-2577.
16. Zhang Shu, Yang Yanmin, Huang Congxin, et al. Stroke prevention in patients with atrial fibrillation in China (2017)[J].Chinese Journal of Arrhythmia, 2018(1):17-30.
17. Mant J, Hobbs FD, Fletcher K, et al. Warfarin versus aspirin for stroke prevention in an elderly community population with atrial fibrillation (the Birmingham Atrial Fibrillation Treatment of the Aged Study, BAFTA): a randomised controlled trial. Lancet.

2007;370(9586):493-503.

18. Chen Wenwen, Dang Heqin, Geng Tao, et al. Evaluation of SAMe-TT2R2 score for predicting the quality of nonvalvular atrial fibrillation using warfarin anticoagulation control[J].Chinese Journal of Hospital Pharmacy,2017,37(020):2072- 2076.
